# Tetra­carbonyldi-μ-chlorido-di­chlorido­bis­(η^5^-cyclo­penta­dien­yl)diirondigallium(2 *Fe—Ga*)

**DOI:** 10.1107/S241431462200832X

**Published:** 2022-08-26

**Authors:** George N. Harakas, Mary Elizabeth Demmin

**Affiliations:** a PO Box 6949, Radford University, Radford, Virginia 24142, USA; Goethe-Universität Frankfurt, Germany

**Keywords:** crystal structure, iron, gallium

## Abstract

The title compound, [Fe_2_Ga_2_(C_5_H_5_)_2_Cl_4_(CO)_4_], has an iron–gallium bond distance of 2.3028 (3) Å. The gallium atoms are connected by two bridging chlorine atoms, each gallium also has one terminal chlorine. The mol­ecule has an inversion center located between the gallium atoms. The cyclo­penta­dienyl ligand is disordered over two sites with an occupancy of 0.57 (2) for the major occupied site.

## Abstract 2

The title compound has an iron–gallium bond distance of 2.3028 (3) Å. The gallium atoms are connected by two bridging chlorine atoms, each gallium also has one terminal chlorine. The mol­ecule has an inversion center located between the gallium atoms. The cyclo­penta­dienyl ligand was modeled for disorder.

## Graphical abstract

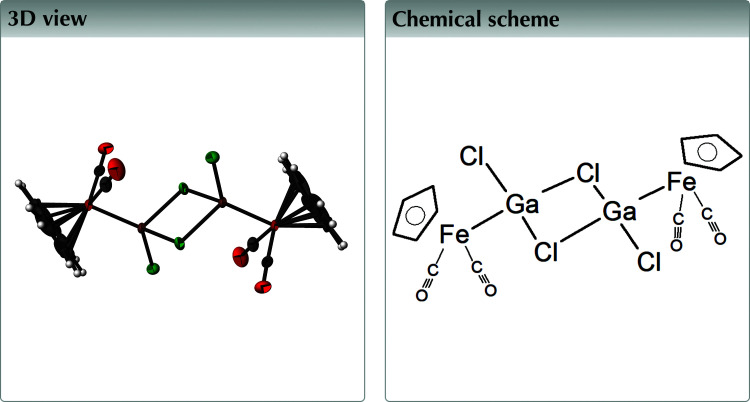

## Structure description

Digallium(II) dichloride has been used in the synthesis of two gallium–ruthenium metal clusters (Harakas & Whittlesey, 1997 ▸). The reaction of Ga_2_Cl_4_·1,4 dioxane with [CpFe(CO)_2_]_2_ in toluene, followed by work-up with a THF, diethyl ether and pentane solution resulted in the isolation of η^5^-CpFeGaCl_2_ *L*, *L* = 1,4-dioxane or THF (Linti *et al.*, 2001 ▸). The reaction of GaCl_3_ and K[CpFe(CO)_2_] in toluene produced [{CpFe(CO)_2_}(Ga(Cl·GaCl_3_)(μ-Cl)]_2_ (Borovik *et al.*, 1999 ▸). In the absence of ether solvents, the reaction of Ga_2_Cl_4_ with [CpFe(CO)_2_]_2_ in toluene produced the title compound, which is a dimeric analog to the compounds isolated by Linti *et al.* (2001 ▸).

**Figure 1:**
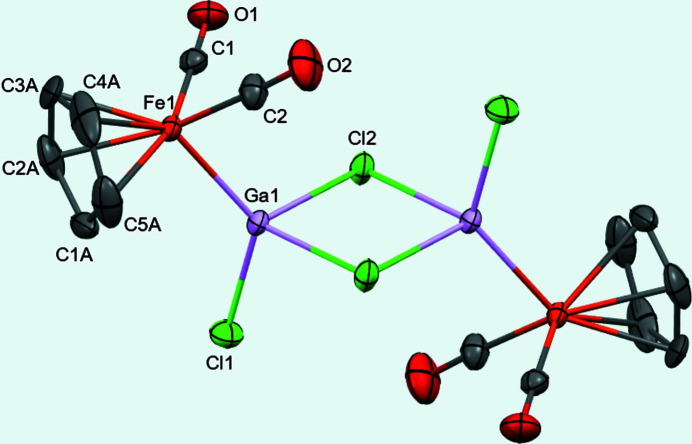
The title compound with 50% displacement ellipsoids. The H atoms and the minor occupied sites of the disordered atoms have been omitted for clarity. Unlabeled atoms are generated by an inversion center.

The Fe1—Ga1 bond distance of 2.3028 (3) Å in the title compound (Fig. 1 ▸) is similar to the 2.317 and 2.316 Å distances found for the etherate compounds (Linti *et al.*, 2001 ▸) but longer than the 2.286 Å value in [{CpFe(CO)_2_}(Ga(Cl·GaCl_3_)(μ-Cl)]_2_ (Borovik *et al.*, 1999 ▸). The gallium–gallium distance of 3.4603 (3) Å is much greater than 2.406 Å for Ga_2_Cl_4_·2 (1,4-dioxane) (Beamish *et al.*, 1979 ▸), indicating there are no metal–metal bonding inter­actions between the gallium atoms.

## Synthesis and crystallization

All manipulations were conducted using inert atmosphere techniques. A stock solution of Ga_2_Cl_4_ was produced by the reaction of Ga (5.496 g, 78.83 mmol) with GaCl_3_ (5.01 g, 28.4 mmol) in 150 ml of toluene. The mixture was heated to reflux for 24 h then cooled to 25°C. In a 150 ml Schlenk flask, [CpFe(CO)_2_]_2_ (1.107 g, 3.128 mmol) in 25 ml of toluene was combined with 25 ml of the Ga_2_Cl_4_ stock solution. The reaction flask was refluxed for 1 h. The mixture was cooled to room temperature, and the solution was deca­nted away from the residue into a new Schlenk flask. Crystals suitable for X-ray analysis formed after 24 h at 25°C. A single crystal was coated with NVH oil and mounted on a MiTeGen loop under a stream of argon gas then cooled to −75°C for data collection.

## Refinement

Crystal data, data collection, and structure refinement details are summarized in Table 1 ▸. The cyclo­penta­dienyl rings were modeled for disorder with two offset ring orientations (C1*A*—C5*A* and C1*B*—C5*B*) at 0.57 (2):0.43 (2) occupancy, respectively.

## Supplementary Material

Crystal structure: contains datablock(s) global, I. DOI: 10.1107/S241431462200832X/bt4125sup1.cif

Structure factors: contains datablock(s) I. DOI: 10.1107/S241431462200832X/bt4125Isup2.hkl

CCDC reference: 2202575

Additional supporting information:  crystallographic information; 3D view; checkCIF report

## Figures and Tables

**Table 1:** Experimental details

Crystal data	
Chemical formula	[Fe_2_Ga_2_(C_5_H_5_)_2_Cl_4_(CO)_4_]
*M*_r_	635.16
Crystal system, space group	Monoclinic, *P*2_1_/*c*
Temperature (K)	198
*a*, *b*, *c* (Å)	8.3567 (3), 7.0331 (2), 16.5792 (6)
β (°)	91.218 (1)
*V* (Å^3^)	974.20 (6)
*Z*	2
Radiation type	Mo *K*α
μ (mm^−1^)	4.76
Crystal size (mm)	0.24 × 0.22 × 0.12

Data collection	
Diffractometer	Bruker D8 Quest Eco, Photon II 7
Absorption correction	Multi-scan (Krause *et al.*, 2015 ▸)
*T*_min_, *T*_max_	0.36, 0.60
No. of measured, independent and observed [*I* > 2σ(*I*)] reflections	100078, 7242, 5892
*R*_int_	0.041
(sin θ/λ)_max_ (Å^−1^)	0.962

Refinement	
*R*[*F* ^2^ > 2σ(*F* ^2^)], *wR*(*F* ^2^), *S*	0.039, 0.086, 1.21
No. of reflections	7242
No. of parameters	164
H-atom treatment	H-atom parameters constrained
Δρ_max_, Δρ_min_ (e Å^−3^)	0.93, −0.99

Computer programs: *APEX3* and *SAINT* (Bruker, 2019 ▸), *SHELXT2018/2* (Sheldrick, 2015*a* ▸), *SHELXL2018/3* (Sheldrick, 2015*b* ▸), and *ShelXle* (Hübschle *et al.*, 2011 ▸).

## full crystallographic data

### Crystal data

**Table:**

[Fe_2_Ga_2_(C_5_H_5_)_2_Cl_4_(CO)_4_]	*F*(000) = 616
*M**_r_* = 635.16	*D*_x_ = 2.165 Mg m^−^^3^
Monoclinic, *P*2_1_/*c*	Mo *K*α radiation, λ = 0.71073 Å
*a* = 8.3567 (3) Å	Cell parameters from 9746 reflections
*b* = 7.0331 (2) Å	θ = 2.9–42.0°
*c* = 16.5792 (6) Å	µ = 4.76 mm^−^^1^
β = 91.218 (1)°	*T* = 198 K
*V* = 974.20 (6) Å^3^	Cube, orange
*Z* = 2	0.24 × 0.22 × 0.12 mm

### Data collection

**Table:**

Bruker D8 Quest Eco, Photon II 7 diffractometer	5892 reflections with *I* > 2σ(*I*)
Detector resolution: 7.3910 pixels mm^-1^	*R*_int_ = 0.041
phi and ω scans	θ_max_ = 43.1°, θ_min_ = 2.5°
Absorption correction: multi-scan (Krause *et al.*, 2015)	*h* = −15→16
*T*_min_ = 0.36, *T*_max_ = 0.60	*k* = −13→13
100078 measured reflections	*l* = −29→31
7242 independent reflections	

### Refinement

**Table:**

Refinement on *F*^2^	0 restraints
Least-squares matrix: full	Hydrogen site location: inferred from neighbouring sites
*R*[*F*^2^ > 2σ(*F*^2^)] = 0.039	H-atom parameters constrained
*wR*(*F*^2^) = 0.086	*w* = 1/[σ^2^(*F*_o_^2^) + (0.0255*P*)^2^ + 0.9013*P*] where *P* = (*F*_o_^2^ + 2*F*_c_^2^)/3
*S* = 1.21	(Δ/σ)_max_ = 0.001
7242 reflections	Δρ_max_ = 0.93 e Å^−^^3^
164 parameters	Δρ_min_ = −0.99 e Å^−^^3^

### Special details

**Table:**

Geometry. All esds (except the esd in the dihedral angle between two l.s. planes) are estimated using the full covariance matrix. The cell esds are taken into account individually in the estimation of esds in distances, angles and torsion angles; correlations between esds in cell parameters are only used when they are defined by crystal symmetry. An approximate (isotropic) treatment of cell esds is used for estimating esds involving l.s. planes.

### Fractional atomic coordinates and isotropic or equivalent isotropic displacement parameters (Å^2^)

**Table:**

	*x*	*y*	*z*	*U*_iso_*/*U*_eq_	Occ. (<1)
Ga1	0.85697 (2)	0.66995 (2)	0.47616 (2)	0.01798 (4)	
Fe1	0.69410 (2)	0.68540 (3)	0.36255 (2)	0.01656 (4)	
Cl1	0.86625 (6)	0.86249 (6)	0.57979 (3)	0.02919 (8)	
Cl2	0.86216 (4)	0.37209 (6)	0.54493 (3)	0.02429 (7)	
O1	0.50545 (19)	0.3758 (2)	0.42638 (10)	0.0335 (3)	
O2	0.9025 (2)	0.4232 (3)	0.28009 (11)	0.0501 (5)	
C1	0.58075 (19)	0.4990 (2)	0.40250 (10)	0.0221 (3)	
C2	0.8235 (2)	0.5277 (3)	0.31382 (11)	0.0281 (3)	
C1A	0.6807 (11)	0.9801 (13)	0.3870 (7)	0.0346 (17)	0.57 (2)
H1A	0.725236	1.046554	0.435902	0.041000*	0.57 (2)
C2A	0.5295 (13)	0.9041 (14)	0.3781 (7)	0.0381 (18)	0.57 (2)
H2A	0.445268	0.906758	0.420085	0.046000*	0.57 (2)
C3A	0.5114 (15)	0.8261 (14)	0.3027 (10)	0.057 (4)	0.57 (2)
H3A	0.411632	0.767194	0.279609	0.068000*	0.57 (2)
C4A	0.653 (2)	0.8541 (18)	0.2625 (4)	0.056 (3)	0.57 (2)
H4A	0.673376	0.817989	0.205359	0.067000*	0.57 (2)
C5A	0.7600 (10)	0.9482 (15)	0.3150 (7)	0.043 (2)	0.57 (2)
H5A	0.871108	0.989832	0.302262	0.052000*	0.57 (2)
C1B	0.627 (4)	0.956 (3)	0.3914 (8)	0.077 (6)	0.43 (2)
H1B	0.628153	1.011381	0.447019	0.092000*	0.43 (2)
C2B	0.4986 (19)	0.865 (3)	0.3545 (15)	0.064 (6)	0.43 (2)
H2B	0.392019	0.842282	0.379064	0.076000*	0.43 (2)
C3B	0.540 (2)	0.8158 (16)	0.2786 (11)	0.050 (4)	0.43 (2)
H3B	0.471033	0.750474	0.237288	0.060000*	0.43 (2)
C4B	0.7000 (17)	0.878 (2)	0.2685 (9)	0.043 (3)	0.43 (2)
H4B	0.763455	0.864982	0.218325	0.052000*	0.43 (2)
C5B	0.7506 (15)	0.9653 (16)	0.3384 (12)	0.052 (4)	0.43 (2)
H5B	0.856690	1.027701	0.348301	0.062000*	0.43 (2)

### Atomic displacement parameters (Å^2^)

**Table:**

	*U*^11^	*U*^22^	*U*^33^	*U*^12^	*U*^13^	*U*^23^
Ga1	0.01699 (6)	0.01791 (7)	0.01880 (7)	0.00328 (5)	−0.00553 (5)	0.00044 (5)
Fe1	0.01504 (7)	0.01978 (9)	0.01474 (8)	0.00420 (6)	−0.00213 (6)	0.00057 (6)
Cl1	0.0355 (2)	0.02587 (18)	0.02603 (18)	0.00044 (15)	−0.00416 (15)	−0.00649 (14)
Cl2	0.01591 (12)	0.02142 (15)	0.03549 (19)	0.00060 (11)	−0.00080 (12)	0.01075 (14)
O1	0.0346 (7)	0.0321 (7)	0.0339 (7)	−0.0102 (6)	0.0019 (5)	−0.0029 (6)
O2	0.0548 (11)	0.0556 (11)	0.0403 (9)	0.0282 (9)	0.0118 (8)	−0.0069 (8)
C1	0.0212 (6)	0.0243 (6)	0.0206 (6)	0.0006 (5)	−0.0028 (5)	−0.0032 (5)
C2	0.0291 (7)	0.0332 (8)	0.0219 (7)	0.0095 (6)	0.0018 (6)	−0.0005 (6)
C1A	0.048 (3)	0.0189 (15)	0.036 (4)	0.005 (2)	−0.022 (3)	−0.0042 (17)
C2A	0.036 (4)	0.027 (3)	0.052 (4)	0.016 (2)	0.017 (3)	0.003 (2)
C3A	0.047 (4)	0.036 (3)	0.085 (9)	0.018 (3)	−0.051 (5)	−0.012 (4)
C4A	0.101 (9)	0.051 (6)	0.017 (2)	0.043 (6)	−0.009 (4)	0.006 (2)
C5A	0.036 (3)	0.033 (4)	0.062 (4)	0.008 (2)	0.020 (3)	0.026 (3)
C1B	0.154 (18)	0.049 (9)	0.026 (3)	0.066 (10)	−0.003 (9)	−0.003 (5)
C2B	0.033 (5)	0.051 (9)	0.108 (15)	0.025 (5)	0.031 (8)	0.050 (8)
C3B	0.067 (8)	0.023 (3)	0.058 (7)	−0.013 (4)	−0.048 (6)	0.017 (4)
C4B	0.050 (5)	0.030 (3)	0.051 (7)	0.011 (3)	0.029 (5)	0.019 (4)
C5B	0.042 (6)	0.022 (2)	0.089 (9)	−0.006 (3)	−0.045 (6)	0.001 (5)

### Geometric parameters (Å, º)

**Table:**

Ga1—Cl1	2.1877 (5)	Fe1—C5A	2.088 (8)
Ga1—Fe1	2.3028 (3)	Fe1—C1A	2.116 (9)
Ga1—Cl2	2.3850 (4)	O1—C1	1.146 (2)
Ga1—Cl2^i^	2.3987 (4)	O2—C2	1.142 (2)
Fe1—C1	1.7555 (17)	C1A—C2A	1.377 (11)
Fe1—C2	1.7578 (18)	C1A—C5A	1.397 (13)
Fe1—C1B	2.045 (12)	C2A—C3A	1.372 (13)
Fe1—C3A	2.057 (8)	C3A—C4A	1.382 (16)
Fe1—C4A	2.062 (8)	C4A—C5A	1.401 (13)
Fe1—C5B	2.066 (11)	C1B—C5B	1.37 (2)
Fe1—C2B	2.068 (11)	C1B—C2B	1.38 (2)
Fe1—C4B	2.069 (11)	C2B—C3B	1.36 (2)
Fe1—C2A	2.083 (9)	C3B—C4B	1.417 (17)
Fe1—C3B	2.086 (11)	C4B—C5B	1.369 (16)
			
Cl1—Ga1—Fe1	128.582 (16)	C2A—Fe1—C5A	64.8 (4)
Cl1—Ga1—Cl2	99.687 (18)	C1—Fe1—C1A	128.9 (3)
Fe1—Ga1—Cl2	115.906 (14)	C2—Fe1—C1A	137.9 (3)
Cl1—Ga1—Cl2^i^	99.924 (18)	C3A—Fe1—C1A	65.1 (3)
Fe1—Ga1—Cl2^i^	116.724 (14)	C4A—Fe1—C1A	65.3 (4)
Cl2—Ga1—Cl2^i^	87.338 (14)	C2A—Fe1—C1A	38.3 (3)
C1—Fe1—C2	92.53 (9)	C5A—Fe1—C1A	38.8 (4)
C1—Fe1—C1B	117.0 (9)	Ga1—Cl2—Ga1^i^	92.662 (14)
C2—Fe1—C1B	150.4 (9)	O1—C1—Fe1	178.04 (15)
C1—Fe1—C3A	98.1 (4)	O2—C2—Fe1	177.2 (2)
C2—Fe1—C3A	122.6 (5)	C2A—C1A—C5A	107.3 (8)
C1—Fe1—C4A	130.6 (5)	C2A—C1A—Fe1	69.6 (5)
C2—Fe1—C4A	95.0 (3)	C5A—C1A—Fe1	69.5 (5)
C3A—Fe1—C4A	39.2 (5)	C3A—C2A—C1A	109.6 (10)
C1—Fe1—C5B	155.8 (6)	C3A—C2A—Fe1	69.6 (5)
C2—Fe1—C5B	111.5 (6)	C1A—C2A—Fe1	72.1 (6)
C1B—Fe1—C5B	39.0 (6)	C2A—C3A—C4A	107.7 (9)
C1—Fe1—C2B	92.8 (4)	C2A—C3A—Fe1	71.7 (5)
C2—Fe1—C2B	148.1 (8)	C4A—C3A—Fe1	70.6 (5)
C1B—Fe1—C2B	39.2 (6)	C3A—C4A—C5A	108.1 (7)
C5B—Fe1—C2B	65.7 (5)	C3A—C4A—Fe1	70.2 (5)
C1—Fe1—C4B	142.8 (5)	C5A—C4A—Fe1	71.3 (4)
C2—Fe1—C4B	92.5 (4)	C1A—C5A—C4A	107.3 (6)
C1B—Fe1—C4B	64.9 (6)	C1A—C5A—Fe1	71.7 (5)
C5B—Fe1—C4B	38.7 (5)	C4A—C5A—Fe1	69.3 (5)
C2B—Fe1—C4B	65.3 (5)	C5B—C1B—C2B	109.0 (10)
C1—Fe1—C2A	98.2 (3)	C5B—C1B—Fe1	71.3 (7)
C2—Fe1—C2A	159.6 (3)	C2B—C1B—Fe1	71.3 (7)
C3A—Fe1—C2A	38.7 (4)	C3B—C2B—C1B	108.4 (12)
C4A—Fe1—C2A	64.9 (4)	C3B—C2B—Fe1	71.6 (7)
C1—Fe1—C3B	104.5 (4)	C1B—C2B—Fe1	69.5 (7)
C2—Fe1—C3B	110.3 (6)	C2B—C3B—C4B	107.0 (11)
C1B—Fe1—C3B	65.1 (5)	C2B—C3B—Fe1	70.2 (7)
C5B—Fe1—C3B	65.9 (4)	C4B—C3B—Fe1	69.4 (7)
C2B—Fe1—C3B	38.2 (6)	C5B—C4B—C3B	108.2 (10)
C4B—Fe1—C3B	39.9 (5)	C5B—C4B—Fe1	70.5 (7)
C1—Fe1—C5A	162.3 (2)	C3B—C4B—Fe1	70.7 (6)
C2—Fe1—C5A	102.4 (3)	C4B—C5B—C1B	107.4 (9)
C3A—Fe1—C5A	65.8 (4)	C4B—C5B—Fe1	70.8 (6)
C4A—Fe1—C5A	39.5 (4)	C1B—C5B—Fe1	69.7 (7)

Symmetry code: (i) −*x*+2, −*y*+1, −*z*+1.
